# Draft Genome Sequence of a Lipolytic Yeast, *Candida aaseri* SH-14

**DOI:** 10.1128/genomeA.00373-17

**Published:** 2017-05-18

**Authors:** Sun Hee Lee, Haeyoung Jeong, Hyeok-Jin Ko, Jung-Hoon Bae, Zool Hilmi Ibrahim, Bong Hyun Sung, Jung-Hoon Sohn

**Affiliations:** a Cell Factory Research Center, Korea Research Institute of Bioscience and Biotechnology (KRIBB), Daejeon, Republic of Korea; b Infectious Disease Research Center, Korea Research Institute of Bioscience and Biotechnology (KRIBB), Daejeon, Republic of Korea

## Abstract

We report here the draft genome sequence of the lipolytic yeast *Candida aaseri* SH-14, isolated from the compost of oil palm empty fruit bunches, and the identification of eight putative lipase genes. This genome information will provide the opportunity to produce potential lipases for a variety of industrial applications.

## GENOME ANNOUNCEMENT

Extracellular microbial lipases (triacylglycerol hydrolase, EC 3.1.1.3) catalyze the hydrolysis of ester bonds between alcohols and carboxylic acids at the lipid-water interface (1). Lipases have been widely used for a broad range of biotechnological and industrial processes (2, 3) and have recently been utilized as an important biocatalyst for the production of biodiesel in the field of bioenergy (4). Although lipases are widely distributed in nature, yeast and fungi have the most commercial potential of the primary sources of lipases used in industrial applications (5). There are various yeasts with lipase-producing capacities; however, only a few have been commercially employed for bulk production (6, 7). Therefore, it is important to isolate and exploit lipolytic yeast species when finding novel lipases for the development of biotechnological and industrial processes. Herein, we present the draft genome sequence of *Candida aaseri* SH-14 as a potential lipase producer.

*Candida aaseri* (syn. *Candida butyri*) SH-14 is a lipolytic yeast isolated from the compost of oil palm empty fruit bunches. We conducted a genome sequencing of *Candida aaseri* SH-14 using the Illumina HiSeq 2500 platform at the Core Facility Management Center at the Korea Research Institute of Bioscience and Biotechnology (KRIBB). We obtained 42.8 million paired-end reads (approximately 400-fold coverage) and assembled them *de novo* using Velvet version 1.2.10, which was facilitated using VelvetOptimiser version 2.2.5 (8). The total size of the draft genome of *C. aaseri* SH-14 was 10,491,190 bp (62 scaffolds including 630 gaps). The *N*_50_ value and the length of the longest contig were 450,417 bp and 1,399,148 bp, respectively. The G+C content was 34.2%, and 126 tRNA-coding sequences were identified using tRNAscan-SE version 2.0 (9). Structural annotation was carried out using the Yeast Genome Annotation Pipeline (YGAP) (10), and Blast2GO was utilized for a functional prediction of the protein-coding sequences (11). As a result, a total of 5,380 proteins were predicted, and we confirmed that 5,037 of them have at least one Gene Ontology (GO) term.

Some *Candida* species, particularly *Candida albicans*, translate the CUG codon as serine instead of leucine; therefore, we have used the Bagheera server (http://www.motorprotein.de/bagheera) to analyze the CUG codon translation of the genes from *C. aaseri* SH-14 (12). The prediction suggested that *C. aaseri* SH-14 belongs to the CUG:Ser group of *Candida* species. We identified eight putative lipase genes from the genome of *C. aaseri* SH-14. Information on putative lipase genes is available from http://genoglobe.kr/kribb/candida_aaseri_2017, where the classification of the putative lipases was based on the oxyanion hole and conserved pentapeptide found in the Lipase Engineering Database (LED) (13, 14).

### Accession number(s).

This whole-genome shotgun project has been deposited at DDBJ/ENA/GenBank under the accession no. LKAN00000000. The version described in this paper is version LKAN01000000.

## References

[1] Jaeger KE, Dijkstra BW, Reetz MT 1999 Bacterial biocatalysts: molecular biology, three-dimensional structures, and biotechnological applications of lipases. Annu Rev Microbiol 53:315–351. doi:10.1146/annurev.micro.53.1.315. 10547694

[2] Jaeger KE, Eggert T 2002 Lipases for biotechnology. Curr Opin Biotechnol 13:390–397. doi:10.1016/S0958-1669(02)00341-5. 12323363

[3] Sharma D, Sharma B, Shukla AK 2011 Biotechnological approach of microbial lipase: a review. Biotechnology 10:23–40. doi:10.3923/biotech.2011.23.40.

[4] Ribeiro BD, de Castro AMd, Coelho MAZ, Freire DMG 2011 Production and use of lipases in bioenergy: a review from the feedstocks to biodiesel production. Enzyme Res 2011:615803. doi:10.4061/2011/615803. 21785707 PMC3137985

[5] Sharma S, Kanwar SS 2014 Organic solvent tolerant lipases and applications. ScientificWorldJournal 2014:625258. doi:10.1155/2014/625258. 24672342 PMC3929378

[6] Treichel H, de Oliveira D, Mazutti MA, Di Luccio M, Oliveira JV 2010 A review on microbial lipases production. Food Bioprocess Technol 3:182–196. doi:10.1007/s11947-009-0202-2.

[7] Gurung N, Ray S, Bose S, Rai V 2013 A broader view: microbial enzymes and their relevance in industries, medicine, and beyond. Biomed Res Int 2013:1–18. doi:10.1155/2013/329121. PMC3784079 24106701

[8] Zerbino DR, Birney E 2008 Velvet: algorithms for *de novo* short read assembly using de Bruijn graphs. Genome Res 18:821–829. doi:10.1101/gr.074492.107. 18349386 PMC2336801

[9] Lowe TM, Eddy SR 1997 tRNAscan-SE: a program for improved detection of transfer RNA genes in genomic sequence. Nucleic Acids Res 25:955–964. doi:10.1093/nar/25.5.0955. 9023104 PMC146525

[10] Proux-Wéra E, Armisén D, Byrne KP, Wolfe KH 2012 A pipeline for automated annotation of yeast genome sequences by a conserved-synteny approach. BMC Bioinformatics 13:237. doi:10.1186/1471-2105-13-237. 22984983 PMC3507789

[11] Conesa A, Gotz S, Garcia-Gomez JM, Terol J, Talon M, Robles M 2005 Blast2GO: a universal tool for annotation, visualization and analysis in functional genomics research. Bioinformatics 21:3674–3676. doi:10.1093/bioinformatics/bti610. 16081474

[12] Mühlhausen S, Kollmar M 2014 Predicting the fungal CUG codon translation with Bagheera. BMC Genomics 15:411. doi:10.1186/1471-2164-15-411. 24885275 PMC4050208

[13] Fischer M, Pleiss J 2003 The Lipase Engineering Database: a navigation and analysis tool for protein families. Nucleic Acids Res 31:319–321. doi:10.1093/nar/gkg015. 12520012 PMC165462

[14] Gupta R, Kumari A, Syal P, Singh Y 2015 Molecular and functional diversity of yeast and fungal lipases: their role in biotechnology and cellular physiology. Prog Lipid Res 57:40–54. doi:10.1016/j.plipres.2014.12.001. 25573113

